# P-1254. Evaluation of Ceftriaxone Population Pharmacokinetics (PK) and Pharmacodynamics (PD) in Critically Ill Pediatric Population

**DOI:** 10.1093/ofid/ofaf695.1445

**Published:** 2026-01-11

**Authors:** Sylwia Marianski, Victor Amajor, Justin Shiau, Amanda Bwint, Anna Sharova, Mark Hall, Nathaniel J Rhodes, Kevin J Downes, Marc H Scheetz

**Affiliations:** Midwestern University, Downers Grove, IL; Children's Hospital of Philadelphia, Philadelphia, Pennsylvania; Midwestern University Pharmacometrics Center of Excellence, Downers Grove, IL; Children's Hospital of Philadelphia, Philadelphia, Pennsylvania; Children's Hospital of Philadelphia, Philadelphia, Pennsylvania; Nationwide Children's Hospital, Columbus, Ohio; Midwestern University, Downers Grove, IL; Children's Hospital of Philadelphia, Philadelphia, Pennsylvania; Midwestern University, Downers Grove, IL

## Abstract

**Background:**

Ceftriaxone (CRO) PKs are not well studied in children with Multiple Organ Dysfunction Syndrome (MODS). We evaluated CRO PK against current CRO breakpoints (PD).

**Figure:**
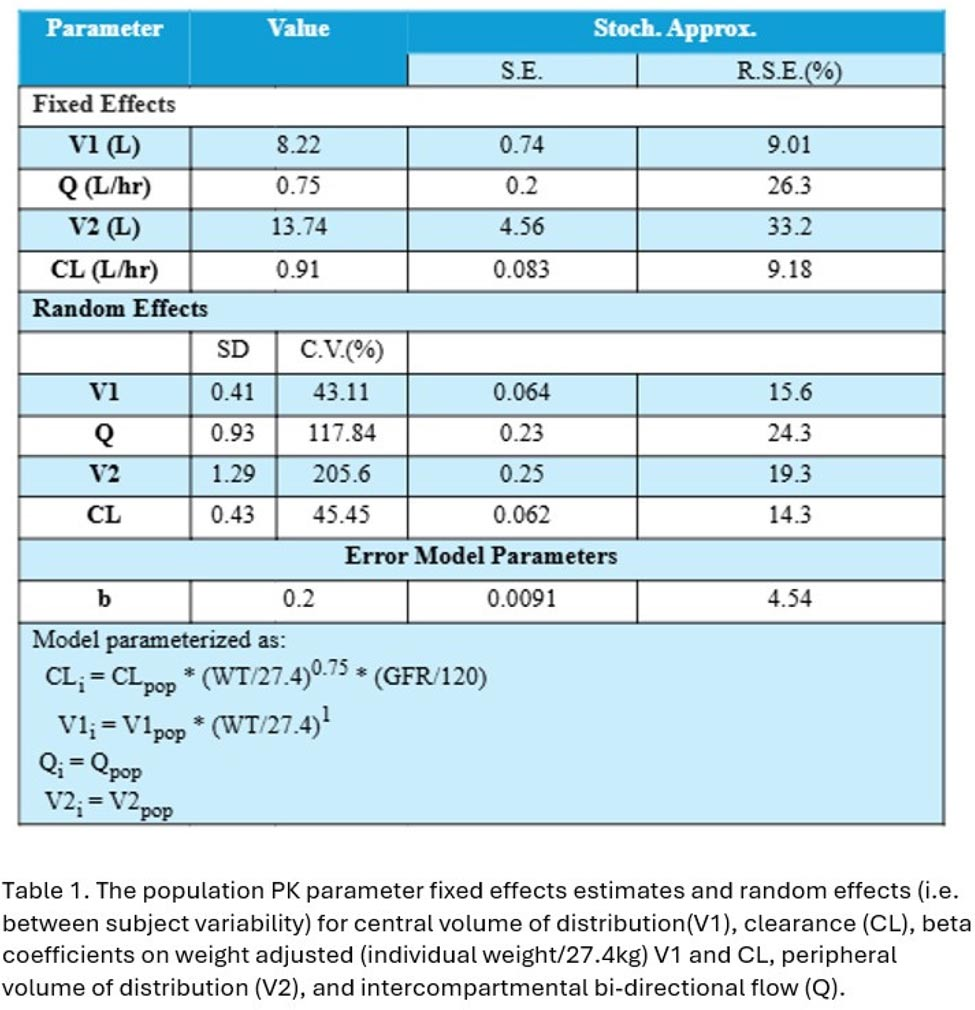

**Figure:**
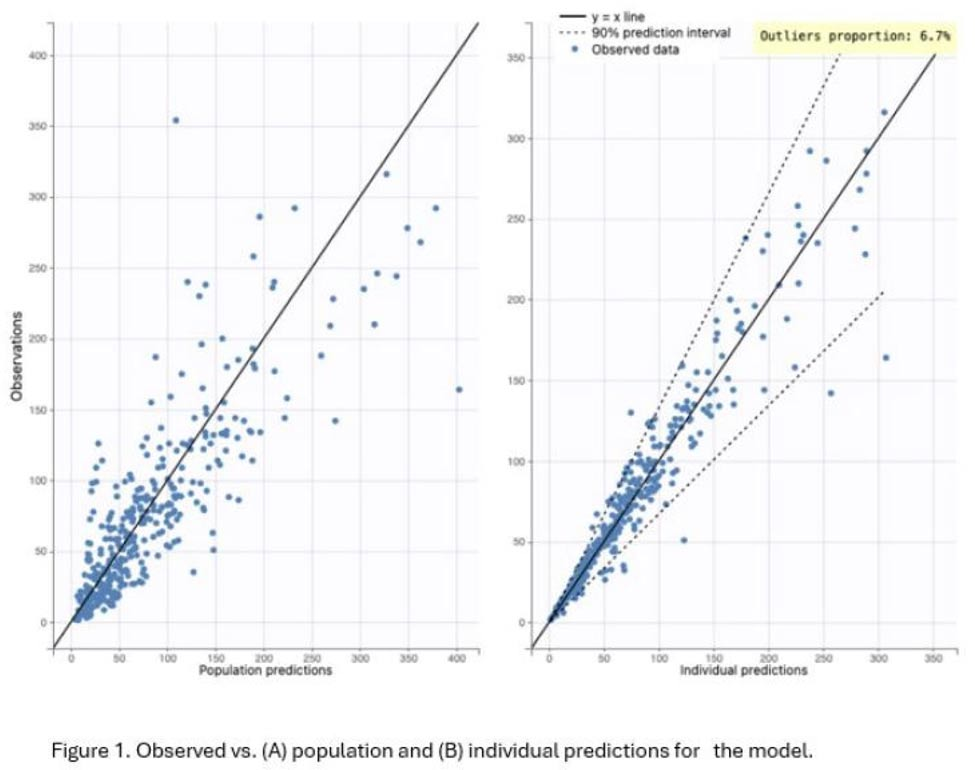

**Methods:**

This is a multi-center prospective study (R01HD103755) of antibiotic PK in critically ill children (< 18 years) with MODS (≥ 2 organ failures) prescribed CRO; those on extracorporeal support were excluded. Up to 15 PK samples were collected over 3 days using volumetric absorptive microsampling (VAMS; 20 µL/sample). Covariate data were collected. CRO was quantified in whole blood VAMS using a validated LC-MS/MS assay. Population PK modeling was performed using Monolix 2024R1. One- and two-compartment models were tested. Fixed allometric scaling of clearance (CL) and central volume of distribution (V1) defined the base models. Other covariates (e.g., Chronic Kidney Disease in Children Under 25 eGFR or U25) were evaluated for inclusion based on 1) the corrected Bayesian Information Criterion 2) reductions in between subject variability (CV%) and 3) and physiological relevance. First-24 hr patient-level PK exposures were calculated using Empirical Bayes Estimates from exact dosing and covariate histories. A literature-based free fraction of 10% was applied. The percentage of time wherein free drug concentrations exceeded the minimum inhibitory concentration (MIC) or *f*T_>MIC_ was calculated for MICs of 1 (susceptible), 2 (intermediate), and 4-8 mg/L resistant pathogens.

**Figure:**
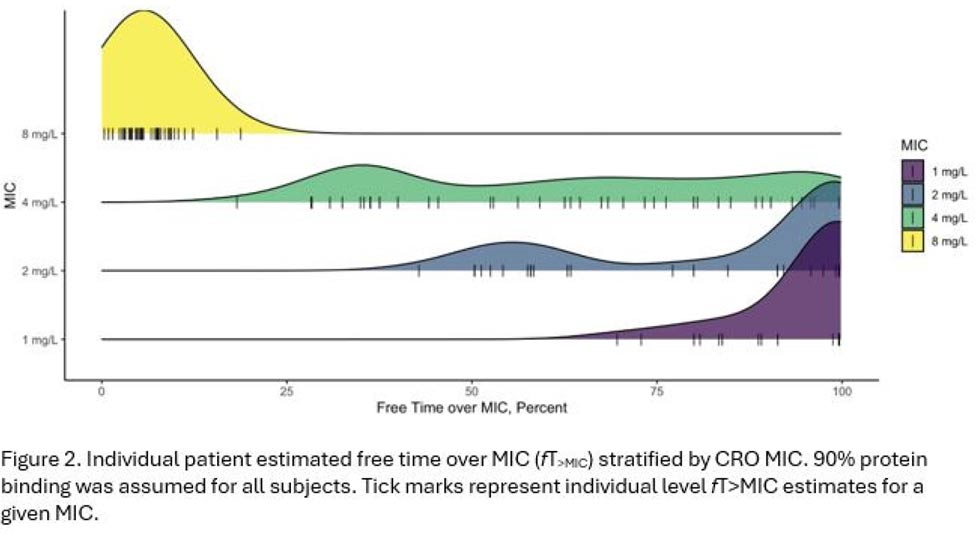

**Results:**

44 patients (2 months to 17 years old) with estimated GFR ranging from 25 to 225 mL/min/1.73 m^2^ (median 100) were enrolled. An allometrically scaled two-compartment model with clearance adjusted based on eGFR sufficiently described the data (Figure 1). Between subject variation (CV%) for V1 and CL was 43.1% and 45.5%, respectively (Table 1). *f*T_>MIC_ was >50% at CRO MICs ≤1 mg/L, variable for CRO MICs of 2-4 mg/L, and < 50% at 8 mg/L (Figure 2).

**Conclusion:**

CRO concentrations in critically ill children with MODS displayed significant variability, even after allometric scaling and accounting for renal function estimates. Variable and low *f*T_>MIC_ (values less than 50%) were seen in MICs >1 mg/L. These data indicate that therapeutic drug monitoring approaches should be developed and are needed for all MICs ≥2 mg/L.

**Disclosures:**

Mark Hall, MD FCCM, Abbvie: Advisor/Consultant|Kiadis: Licensing income unrelated to this submission|Partner Therapeutics: Partner Therapeutics provides study drug for a clinical trial for which I am PI. This trial is unrelated to the submitted abstract|Sobi: Sobi provides study drug for a clinical trial for which I am PI. This trial is unrelated to the submitted abstract Nathaniel J. Rhodes, PharmD MS, Apothecademy, LLC: Advisor/Consultant Kevin J. Downes, MD, Paratek Pharmaceuticals, Inc.: Grant/Research Support|Veloxis Pharmaceuticals, Inc.: Grant/Research Support Marc H. Scheetz, PharmD, MSc, Doseme: Advisor/Consultant|other: Additional not relevant to this abstract. If more information is needed about unrelated relationships, I can provide it.

